# Sodium-Glucose Cotransporter 2 Inhibitors and Serious Liver Events in Patients With Cirrhosis

**DOI:** 10.1001/jamanetworkopen.2025.18470

**Published:** 2025-06-27

**Authors:** Mohamad-Noor Abu-Hammour, Rashid Abdel-Razeq, Aravinthan Vignarajah, Raneem Khedraki, Omar T. Sims, Nishanthi Vigneswaramoorthy, Dian J. Chiang

**Affiliations:** 1 Department of Medicine, Cleveland Clinic Foundation, Cleveland, Ohio; 2 Department of Gastroenterology, Hepatology and Nutrition, Cleveland Clinic Foundation, Cleveland, Ohio; 3 Department of Quantitative Health Sciences, Cleveland Clinic Foundation, Cleveland, Ohio; 4 Cleveland Clinic Lerner College of Medicine, Case Western Reserve University School of Medicine, Cleveland, Ohio; 5 Department of Internal Medicine, The State University of New York Upstate Medical University, Syracuse

## Abstract

**Importance:**

Cirrhosis is a significant global health burden, with serious liver-related complications leading to high morbidity and mortality. Effective therapeutic options to mitigate these complications remain limited. Sodium-glucose cotransporter 2 (SGLT-2) inhibitors, primarily used in diabetes and heart failure management, may offer additional liver-related benefits.

**Objective:**

To evaluate the association between SGLT-2 inhibitor use and the risk of serious liver events in patients with cirrhosis receiving furosemide and spironolactone.

**Design, Setting, and Participants:**

This cohort study used data from over 120 health care organizations within the TriNetX platform. Adult patients with cirrhosis who were receiving furosemide and spironolactone from January 2013 to July 2021 were included. Patients who were receiving SGLT-2 inhibitors plus furosemide and spironolactone were matched with a control group of patients who were receiving furosemide and spironolactone alone according to age, demographics, and comorbidities using 1:1 propensity matching. Each patient was followed up for 3 years; follow-up ended on July 11, 2024.

**Exposure:**

Use of SGLT-2 inhibitors.

**Main Outcomes and Measures:**

The primary outcome was a composite of serious liver events defined as incidence of ascites, variceal development, hyponatremia, or all-cause mortality. Secondary outcomes included incidence of variceal bleeding, paracentesis, spontaneous bacterial peritonitis, hepatic encephalopathy, hepatorenal syndrome, hepatocellular carcinoma, hypoglycemia, and all-cause hospitalizations. Continuous variables were compared using an independent-samples *t* test; categorical variables were compared using the Pearson χ^2^ test.

**Results:**

Among 10 660 propensity-matched patients (mean [SD] age, 63.8 [10.7] years; 57.8% male), those receiving SGLT-2 inhibitors had a lower incidence of serious liver events compared with control patients (hazard ratio [HR], 0.68 [95% CI, 0.66-0.71]; *P* < .001). Secondary outcomes included hepatorenal syndrome (HR, 0.47 [95% CI, 0.40-0.56]), spontaneous bacterial peritonitis (HR, 0.55 [95% CI, 0.46-0.65]), paracentesis (HR, 0.54 [95% CI, 0.50-0.60]), variceal bleeding (HR, 0.79 [95% CI, 0.73-0.84]), hypoglycemia (HR, 0.75 [95% CI, 0.62-0.91]), and all-cause hospitalizations (HR, 0.67 [95% CI, 0.63-0.71]), all of which were associated with a reduced risk among those in the SGLT-2 inhibitors group.

**Conclusions and Relevance:**

In this cohort study of adults with cirrhosis who were receiving diuretic therapy, the findings suggest that SGLT-2 inhibitor use was associated with a lower incidence of serious liver events. These findings further suggest a potential role for SGLT-2 inhibitors in cirrhosis management.

## Key Points

**Question:**

Is there an association between sodium-glucose cotransporter 2 (SGLT-2) inhibitors and serious liver events in patients with cirrhosis who receive diuretic therapy?

**Findings:**

In this cohort study of 10 660 patients with cirrhosis who were receiving furosemide and spironolactone, use of SGLT-2 inhibitors was associated with a significantly reduced risk of serious liver events compared with diuretic therapy alone.

**Meaning:**

These findings suggest that SGLT-2 inhibitors are potentially beneficial in treating patients with cirrhosis who are receiving diuretic therapy and may offer liver-related benefits.

## Teaser

This cohort study evaluates whether there is an association between sodium-glucose cotransporter 2 (SGLT-2) inhibitor use and risk of serious liver events among patients with cirrhosis who are being treated with furosemide and spironolactone.

## Introduction

Sodium-glucose cotransporter 2 (SGLT-2) inhibitors are a class of antihyperglycemic agents that act by inhibiting the SGLT proteins in the proximal convoluted tubules of the kidney.^1^ By inhibiting this protein, SGLT-2 inhibitors limit the reabsorption of filtered sodium and glucose, thereby enhancing urinary glucose excretion, natriuresis, and improving urine output.^2,3^ Beyond their primary role in managing type 2 diabetes, SGLT-2 inhibitors have demonstrated significant cardiovascular benefits, including reductions in cardiovascular mortality and hospitalizations. These benefits are largely associated with their diuretic effects and subsequent decrease in activation of the renin-angiotensin-aldosterone system (RAAS).^4^ By lowering RAAS activity, SGLT-2 inhibitors help improve hemodynamic stability and reduce myocardial stress, which has led to their recommendation in clinical practice guidelines for patients with heart failure, irrespective of ejection fraction or diabetes status.^5^

Liver cirrhosis, characterized by advanced liver fibrosis and progressive hepatic dysfunction, represents a global health burden, ranking as the 11th-most common cause of mortality worldwide. Diuretic therapies have demonstrated benefits in controlling ascites in patients with cirrhosis.^6,7^ However, approximately 10% of patients with cirrhosis develop refractory ascites, which are defined as ascites that cannot be mobilized or recur early despite optimal diuretic therapy. These patients often require large-volume paracentesis as a mainstay of treatment,^8^ highlighting the need for more effective and innovative therapies to improve patient outcomes and quality of life.

In the context of patients with cirrhosis, who are treated by diuretic therapy such as spironolactone and furosemide, the RAAS-modulating effects of SGLT-2 inhibitors could theoretically provide additional benefits. These benefits include improved volume status without exacerbating electrolyte imbalances, which is particularly relevant given the hemodynamic challenges in this patient population. The natriuretic and diuretic effects of SGLT-2 inhibitors could help manage fluid overload while potentially reducing the need for large-volume paracentesis. Given the lack of current data on the safety or efficacy of SGLT-2 inhibitors in patients with cirrhosis, we hypothesized that these agents were associated with a reduced risk of serious liver events in patients with cirrhosis on diuretic therapy.

## Methods

### Study Design and Propensity Score Matching

This retrospective cohort study was conducted using data from TriNetX, a multi-institutional health research network. Using the TriNetX platform, we accessed deidentified electronic health records from over 212 million patients across 120 major health care organizations.^9^ The built-in analytic functions of TriNetX enable patient-level analyses while ensuring that only population-level data are reported.

This study was approved by WCG Clinical, which granted a waiver to TriNetX as a federated network and was deemed exempt from informed consent owing to the use of existing, non–human participant data that were deidentified per the US Health Insurance Portability and Accountability Act privacy rule. The study followed the Strengthening the Reporting of Observational Studies in Epidemiology (STROBE) reporting guideline.

We included patients with cirrhosis (*International Statistical Classification of Diseases and Related Health Problems, Tenth Revision* [*ICD-10*] codes K74.6 and K74.69), who were taking furosemide (RxNorm [National Library of Medicine] code 4603) and spironolactone (RxNorm code 9997) between January 2013 and July 2021. For patients receiving an SGLT-2 inhibitor (Anatomical Therapeutic Chemical code A10BK), the index event was defined as the date on which they were concurrently prescribed spironolactone, furosemide, and an SGLT-2 inhibitor. For the control group, the index event was the date on which they were prescribed concurrent spironolactone and furosemide but not an SGLT-2 inhibitor. Each patient was followed up for 3 years from the index event, with follow-up ending on July 11, 2024. Patients were excluded if they were younger than 19 years, were undergoing dialysis (*Current Procedural Terminology* [*CPT*] code 1012740), had a history of kidney (*CPT* code 1008098) or liver (*CPT* code 1007811) transplant, or had a history of hepatocellular carcinoma (HCC; *ICD-10* code C22.0).

To mitigate confounding bias and to enhance validity, patients in the SGLT-2 inhibitors–user cohort were matched to patients in the control group using 1:1 propensity scores generated by using greedy nearest-neighbor algorithms with a caliper width of 0.1. Covariates in the propensity score model included demographics (age and race and ethnicity), comorbidities (hypertension, ischemic heart diseases, heart failure, chronic obstructive pulmonary disease, type 2 diabetes, cerebral infarction, and chronic kidney disease), medications (nonselective β-blocker [NSBB], pantoprazole, and omeprazole), and laboratory values (total bilirubin, albumin, international normalized ratio [INR], creatinine, sodium, aspartate aminotransferase, and alanine aminotransferase). Race and ethnicity (including American Indian or Alaska Native, Asian, Black or African American, Native Hawaiian or Other Pacific Islander, or White) were abstracted from electronic health records and were included to examine potential disparities in outcomes.

### Exposure and Outcomes

The primary exposure was the use of SGLT-2 inhibitors. The primary outcome was serious liver events, a composite variable comprised of the incidence of esophageal (*ICD-10* code I85) or gastric (*ICD-10* code I86.4) variceal development, ascites (*ICD-10* code R18.8), hyponatremia, or all-cause mortality. Secondary outcomes included all-cause hospitalizations (*CPT* code 1013659), hepatic encephalopathy (*ICD-10* code K76.8), hepatorenal syndrome (*ICD-10* code K76.7), spontaneous bacterial peritonitis (*ICD-10* code K65.2), paracentesis (*CPT* code 1020907), variceal bleeding (*ICD-10* code I85.01), HCC (*ICD-10* code C22.0), and hypoglycemia (*ICD-10* code E16.2). Outcome measures were based on *ICD-10* and *CPT* codes.

### Statistical Analysis

Propensity score matching was used to balance baseline characteristics between patients who used SGLT-2 inhibitors and those who did not. Continuous variables are expressed as means (SDs) and were compared using an independent-samples *t* test; categorical variables are expressed as frequency distributions and were compared using the Pearson χ^2^ test. A standard mean difference of less than 0.1 indicated negligible differences in covariates between groups.^10,11^ For each outcome, we performed Cox proportional hazards regression modeling to calculate hazard ratios (HRs), ensuring that censoring and potential confounding factors were appropriately accounted for. A 2-sided α < .05 was set as the threshold for statistical significance. All analyses were conducted in real time using the TriNetX research platform.^9^

#### Subgroup Analysis

Due to the heterogeneous nature of cirrhosis and to assess the robustness of our findings, we conducted a prespecified subgroup analysis by a Model for End-Stage Liver Disease (MELD) 3.0 score (where scores range from 6 to 40, with higher scores indicating severe liver disease). As TriNetX does not provide precomputed MELD 3.0 scores, we imposed baseline laboratory thresholds aligned with a MELD 3.0 score of 15 or less by restricting the relevant variables (eg, total bilirubin, INR, and creatinine) to their upper limits of normal. This approach ensured that all patients included in this subgroup analysis had an equivalent MELD 3.0 score of 15 or less, thereby representing a more compensated cirrhotic population. Separate propensity scores were estimated for each subgroup, and 2-sided *P* values of <.05 were considered statistically significant.

#### Sensitivity Analysis

Additionally, we conducted sensitivity analyses using E-values for the calculated odds ratios. An E-value quantifies the minimum strength of association that an unmeasured confounder would need to have with both the exposure and the outcome to nullify an observed association. This allows us to assess the extent to which our results might be influenced by potential unmeasured confounding factors. We followed the method described by VanderWeele and Ding for calculating E-values.^12^ E-values greater than 2 indicate that an unmeasured confounder would need to be strong to completely negate the observed association, suggesting that our findings are robust to potential unmeasured confounding. Conversely, E-values closer to 1 imply that even a weak confounder could potentially explain the observed effects, indicating that those associations are less robust.

Furthermore, we used falsification testing to further validate our findings and mitigate the possible influence of unmeasured confounders. This approach follows the method advocated by Prasad and Jena,^13^ who recommend using such end points to validate true observational associations and ensure that the observed associations are not artifacts of unmeasured confounding.

## Results

### Study Sample Characteristics

The study sample included 118 751 patients before propensity score matching; of these, 5330 patients used SGLT-2 inhibitors and 113 421 (the control group) did not. After propensity score matching, 10 660 patients (mean [SD] age, 63.8 [10.7] years; 37.2% female and 57.8% male) were included in the study (eFigure in Supplement 1); of these, 5330 used SGLT-2 inhibitors and 5330 (the control group) did not. Using standardized mean differences, covariate means between the SGLT-2 inhibitor group and the control group were alike except for aspartate aminotransferase, INR, total bilirubin, and albumin (standardized mean differences, >0.01 for all). In terms of race and ethnicity, 66 patients (0.6%) were American Indian or Alaska Native, 430 (4.0%) were Asian, 1200 (11.3%) were Black or African American, 73 (0.7%) were Native Hawaiian or Other Pacific Islander, 7063 (66.3%) were White, and 1828 (17.2%) were of unspecified race or ethnicity in the TriNetX dataset (Table).

**Table.** Baseline and Clinical Characteristics of the Study Cohort^a^

Characteristic	Before propensity score matching (N = 118 751)			After propensity score matching (n = 10 660)		
	SGLT-2 inhibitor group (n = 5330)	Control group (n = 113 421)	Standardized mean difference	SGLT-2 inhibitor group (n = 5330)	Control group (n = 5330)	Standardized mean difference
Age, mean (SD), y	63.8 (10.7)	60.3 (12.1)	0.307	63.8 (10.7)	64.1 (11.1)	0.028
Sex						
Female	1984 (37.2)	45 657 (40.2)	0.077	1984 (37.2)	1977 (37.1)	0.003
Male	3083 (57.8)	61 884 (54.6)	0.046	3083 (57.8)	3072 (57.6)	0.004
Unspecified	263 (4.9)	5880 (5.2)	NA	263 (4.9)	281 (5.3)	NA
Race and ethnicity						
American Indian or Alaska Native	33 (0.6)	578 (0.5)	0.008	33 (0.6)	35 (0.7)	0.010
Asian	215 (4.3)	7575 (6.7)	0.110	215 (4.3)	202 (3.8)	0.025
Black or African American	600 (11.4)	9137 (8.0)	0.107	600 (11.4)	594 (11.1)	0.007
Native Hawaiian or Other Pacific Islander	35 (0.6)	323 (0.3)	0.053	35 (0.6)	38 (0.7)	0.007
White	3498 (65.6)	73 202 (64.5)	0.002	3498 (65.6)	3565 (66.9)	0.027
Unspecified	949 (17.8)	22 606 (19.9)	NA	949 (17.8)	896 (16.8)	NA
BMI, mean (SD)	31.8 (7.7)	29.7 (7.6)	0.278	31.8 (7.7)	31.4 (8.0)	0.048
Comorbidity^b^						
Essential hypertension	4354 (81.7)	55 263 (49.6)	0.718	4354 (81.7)	4378 (82.1)	0.012
Ischemic heart diseases	2986 (56.0)	24 776 (22.2)	0.738	2986 (56.0)	2952 (55.4)	0.013
Heart failure	3037 (57.0)	23 023 (20.7)	0.803	3037 (57.0)	3064 (57.5)	0.010
Chronic obstructive pulmonary disease	1266 (23.8)	14 825 (13.3)	0.271	1266 (23.8)	1180 (22.1)	0.038
Cerebral infarction	484 (9.1)	4063 (3.6)	0.224	484 (9.1)	447 (8.4)	0.025
Type 2 diabetes	4246 (79.7)	39 162 (35.2)	1.008	4246 (79.7)	4302 (80.7)	0.026
Chronic kidney disease	2407 (45.2)	19 675 (17.7)	0.620	2407 (45.2)	2262 (42.4)	0.055
Medication^c^						
β-Blockers	4473 (83.9)	55 259 (49.6)	0.782	4473 (83.9)	4485 (81.1)	0.006
Pantoprazole	3597 (67.5)	46 958 (42.2)	0.526	3597 (67.5)	3511 (65.9)	0.077
Omeprazole	2159 (40.5)	27 267 (24.5)	0.347	2159 (40.5)	2082 (39.1)	0.030
Laboratory values, mean (SD)						
Alanine aminotransferase, U/L	32.1 (44.7)	43.8 (74.2)	0.190	32.1 (44.7)	36.0 (57.8)	0.075
Aspartate aminotransferase, U/L	39.4 (46.5)	71.5 (120.6)	0.351	39.4 (46.5)	51.5 (90.7)	0.168
INR, mg/dL	1.3 (0.5)	1.4 (0.6)	0.230	1.3 (0.5)	1.4 (0.6)	0.186
Bilirubin, total, mg/dL	1.4 (3.5)	2.9 (4.6)	0.375	1.4 (3.5)	1.8 (3.5)	0.126
Albumin, g/dL	3.6 (0.7)	3.1 (0.7)	0.801	3.6 (0.7)	3.2 (0.7)	0.541
Creatinine, mg/dL	1.6 (6.7)	1.1 (2.9)	0.079	1.6 (6.7)	1.4 (3.1)	0.032
Sodium, mEq/L	137.2 (4.3)	136.5 (4.7)	0.142	137.2 (4.3)	137.1 (4.5)	0.023

Abbreviations: BMI, body mass index (calculated as weight in kilograms divided by height in meters squared); INR, international normalized ratio; NA, not applicable; SGLT-2, sodium-glucose cotransporter 2.

SI conversion factors: To convert alanine aminotransferase to μkat/L, multiply by 0.0167; aspartate aminotransferase to μkat/L, multiply by 0.0167; bilirubin, total to μmol/L, multiply by 17.104; albumin to g/L, multiply by 10; creatinine to μmol/L, multiply by 88.4; and sodium to mmol/L, multiply by 1.0.

^a^ Data are presented as No. (%) of patients unless otherwise indicated. Percentages may not sum to 100% owing to rounding.

^b^ A given patient could have more than 1 comorbidity.

^c^ A given patient could be taking more than 1 medication.

### Primary and Secondary Outcomes

**Figure 1.**
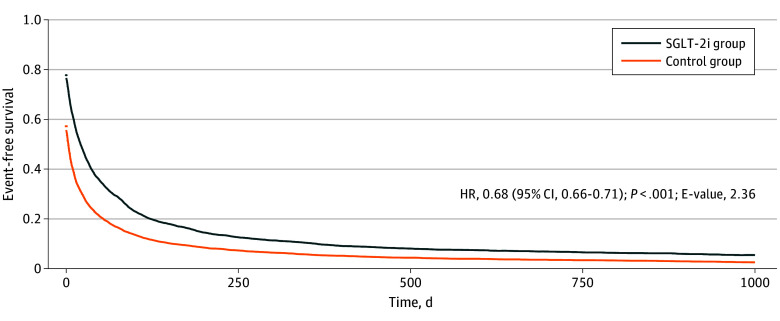
Event-Free Survival of Serious Liver Events by Study Group HR indicates hazard ratio; SGLT-2i, sodium-glucose cotransporter 2 inhibitor.

The study’s main outcome was the incidence of serious liver events (ie, a composite variable comprised of ascites, esophageal or gastric varices with or without bleeding, hyponatremia, or all-cause mortality). Compared with furosemide and spironolactone alone, use of SGLT-2 inhibitors with concurrent furosemide and spironolactone was associated with a statistically significant reduction in the risk of serious liver events (HR, 0.68 [95% CI, 0.66-0.71]; E-value, 2.36) (Figure 1).

**Figure 2.**
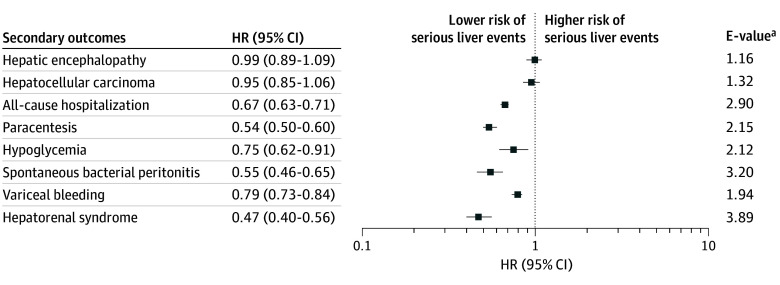
Secondary Outcomes Analysis HR indicates hazard ratio. ^a^E-values quantify the minimum strength of association that an unmeasured confounder would need to have with both the exposure and the outcome to nullify an observed association.

The secondary outcomes’ analysis revealed that the use of SGLT-2 inhibitors was associated with a reduced risk of cirrhosis complications (Figure 2). Specifically, patients taking SGLT-2 inhibitors were associated with a lower risk of hepatorenal syndrome (HR, 0.47 [95% CI, 0.40-0.56]; E-value, 3.89), variceal bleeding (HR, 0.79 [95% CI, 0.73-0.84]; E-value, 1.94), spontaneous bacterial peritonitis (HR, 0.55 [95% CI, 0.46-0.65]; E-value, 3.20), paracentesis (HR, 0.54 [95% CI, 0.50-0.60]; E-value, 2.15), hypoglycemia (HR, 0.75 [95% CI, 0.62-0.91]; E-value, 2.12), and all-cause hospitalizations (HR, 0.67 [95% CI, 0.63-0.71]; E-value, 2.90). However, there was no association observed between use of SGLT-2 inhibitors and hepatic encephalopathy (HR, 0.99 [95% CI, 0.89-1.09]; E-value 1.16) and HCC (HR, 0.95 [95% CI, 0.85-1.06]; E-value, 1.32) between the 2 groups.

### Subgroup Analysis

The primary objective of the subgroup analysis was to assess whether significant clinical outcomes could be observed in patients with MELD 3.0 scores of 15 or less. To establish this, we set parameters for the MELD 3.0 variables at their upper limits of normal. Theoretically, a patient with all variables (albumin, total bilirubin, sodium, creatinine, and INR) at their upper limit of normal would have a MELD 3.0 score of 15 or less. Outcomes were compared between patients who used SGLT-2 inhibitors and those who did not.

**Figure 3.**
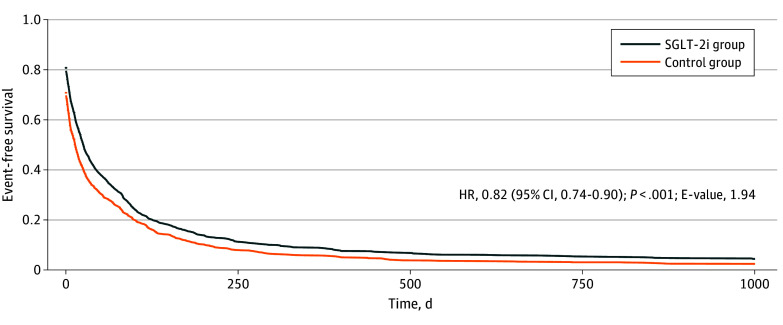
Event-Free Survival of Serious Liver Events Among Patients With a Model for End-Stage Liver Disease (MELD) 3.0 Score of 15 or Less MELD 3.0 scores range from 6 to 40, with higher scores indicating severe liver disease. HR indicates hazard ratio; SGLT-2i, sodium-glucose cotransporter 2 inhibitor.

In the subgroup analysis, use of SGLT-2 inhibitors was also associated with a decreased incidence of serious liver events in patients with MELD 3.0 scores of 15 or less (HR, 0.82 [95% CI, 0.74-0.90]; *P* < .001; E-value, 1.94) (Figure 3). Similarly, SGLT-2 inhibitors were statistically associated with reduced risk of paracentesis (HR, 0.67 [95% CI, 0.47-0.96]; E-value, 2.51) and all-cause hospitalizations (HR, 0.86 [95% CI, 0.74-0.99]; E-value, 1.53). However, there were no associations between use of SGLT-2 inhibitors and incidence of variceal bleed (HR, 0.98 [95% CI, 0.82-1.19]; E-value, 1.3), hepatic encephalopathy (HR, 1.35 [95% CI, 1.00-1.83]; E-value, 1.81), hepatorenal syndrome (HR, 0.41 [95% CI, 0.15-1.15]; E-value, 1.93), spontaneous bacterial peritonitis (HR, 1.01 [95% CI, 0.51-2.01]; E-value, 1.32), or hypoglycemia (HR, 0.68 [95% CI, 0.39-1.18]; E-value, 2.85).

### Sensitivity Analysis

To ensure that the associations observed did not occur due to unmeasured confounding, we conducted sensitivity analyses using E-values for the calculated odds ratios in addition to falsification tests. E-values for the primary and several secondary outcomes suggest that unmeasured confounding would be unlikely to negate the observed associations, particularly for the risk of serious liver events.

To further validate our results, we performed falsification tests by examining the incidence of unrelated outcomes—acute gastroenteritis and osteoporosis—among patients taking SGLT-2 inhibitors. Following propensity score matching, there was no association in the incidence of these falsification end points between the SGLT-2 inhibitors group and the control group. Specifically, acute gastroenteritis was reported in 44 patients receiving SGLT-2 inhibitors and 48 control patients (HR, 0.97 [95% CI, 0.64-1.46]; E-value, 1.41), while osteoporosis occurred in 74 patients receiving SGLT-2 inhibitors compared with 71 patients in the control group (HR, 1.09 [95% CI, 0.79-1.51]; E-value, 1.25).

## Discussion

In this large, multi-institutional cohort study of 120 health care organizations that included over 118 000 patients, the use of SGLT-2 inhibitors in patients with cirrhosis taking furosemide and spironolactone was associated with significantly lower rates of our composite outcome of serious liver events compared with patients with cirrhosis who did not receive SGLT-2 inhibitors. Additionally, these agents were associated with a reduced incidence of spontaneous bacterial peritonitis, hepatorenal syndrome, variceal bleeding, paracentesis, hypoglycemia, and all-cause hospitalizations. While recent studies have highlighted the benefits of SGLT-2 inhibitors in patients with heart failure and diabetes, our findings extend these potential benefits to include liver-related complications in a population with cirrhosis.

Recent evidence strongly supports the potential therapeutic value of SGLT-2 inhibitors in patients with advanced liver disease, demonstrating improvements in both liver-related outcomes and overall survival. In a large propensity-matched cohort, patients with type 2 diabetes and cirrhosis receiving dual therapy (metformin plus an SGLT-2 inhibitor) showed significantly lower 5-year mortality, decreased incidence of decompensated cirrhosis, and a reduction in HCC incidence compared with those taking metformin alone.^14^ Similarly, a population-based retrospective study on patients with type 2 diabetes and metabolic dysfunction-associated steatotic liver disease (MASLD; formerly nonalcoholic fatty liver disease [NAFLD]) reported that SGLT-2 inhibitor use was associated with a decreased incidence of developing cirrhosis and HCC.^15^ We theorize that the observed associated benefits of SGLT-2 inhibitors in patients with cirrhosis may be attributed to their capacity for net free water excretion, resulting from glucosuria and osmotic diuresis, in addition to their RAAS-modulating effects reported in previous studies.^2,3,4^ Notably, our study demonstrated a significantly reduced risk of ascites among patients with cirrhosis treated with SGLT-2 inhibitors. Supporting this observation, a recent Japanese case report details the case of a 59-year-old patient with refractory ascites due to decompensated liver cirrhosis who experienced marked ascites reduction following SGLT-2 inhibitor initiation for uncontrolled diabetes.^16^

Patients with cirrhosis and ascites often present with concurrent hyponatremia, with sodium levels significantly associated with the prognosis of decompensated liver disease.^17,18^ SGLT-2 inhibitors may mitigate hyponatremia through sodium-independent water excretion driven by osmotic diuresis. For patients with impaired free water excretion, such as those with syndrome of inappropriate antidiuresis, SGLT-2 inhibitors have shown particular benefit.^19^ A randomized, double-blind, placebo-controlled crossover trial demonstrated that treatment with empagliflozin, an SGLT-2 inhibitor, significantly increased serum sodium levels in patients with chronic syndrome of inappropriate antidiuresis-induced hyponatremia.^19^ Although SGLT-2 inhibitors induce natriuresis, compensatory mechanisms in the distal tubules prevent sustained natriuresis, leading to stable or gradually increasing serum sodium concentrations.

One recent study focused on studying the effects of SGLT-2 inhibitors on patients with diabetes with MASLD and hepatic fibrosis using the Fibrosis-4 index, revealing a significant reduction in hepatic fibrosis following 12 months of treatment with SGLT-2 inhibitors in patients with a high baseline Fibrosis-4 index. This finding suggests that SGLT-2 inhibitors may play a role in improving outcomes in this patient population.^20^ Although MASLD represents a distinct etiology, the observed reduction in hepatic fibrosis suggests that SGLT-2 inhibitors may have beneficial effects on the liver that could extend to patients with cirrhosis. Proposed mechanisms include inhibition of proinflammatory cytokines such as interleukin-6, tumor necrosis factor-α, and monocyte chemoattractant protein-1, all of which contribute to the progression of chronic liver disease.^21,22^

Our subgroup analysis focused on patients with cirrhosis with MELD 3.0 scores of 15 or less to analyze a relatively more compensated population with cirrhosis and to assess whether our outcomes differed in this subgroup. Results indicated that SGLT-2 inhibitors were associated with a decreased incidence of serious liver events, paracentesis, and all-cause hospitalizations, suggesting potential benefits even in patients with relatively preserved liver function. Although there were no associations between use of SGLT-2 inhibitors and other cirrhosis complications—such as variceal bleeding, hepatic encephalopathy, hepatorenal syndrome, spontaneous bacterial peritonitis, and hypoglycemia—in this subgroup, we can attribute this to the relatively compensated status of this subpopulation.

It is important to note that, although not statistically significant, the HR for hepatic encephalopathy was greater than 1 (HR, 1.35 [95% CI, 1.00-1.83]; E-value, 1.81) in our subgroup analysis. This is consistent with findings from a previous trial on biliary cirrhotic rats, which demonstrated that empagliflozin exacerbated hepatic encephalopathy, as evidenced by decreased motor activity,^23^ suggesting that SGLT-2 inhibitors should be used with caution in patients with cirrhosis who are at an increased risk for hepatic encephalopathy.

### Limitations

Our study has notable limitations. We acknowledge the potential for misclassification bias, as our study relied on *ICD-10* codes to define outcomes, which may not have perfectly captured the complexity of cirrhotic complications. To mitigate this risk, we performed rigorous sensitivity analyses, including falsification testing with unrelated outcomes such as osteoporosis and acute gastroenteritis. These tests showed no significant differences between the 2 cohorts, which reinforces the robustness of our findings. The retrospective design of the study may have introduced confounding, selection, and information bias, which could have impacted the results. Despite the use of propensity score matching and E-value analyses, these confounding variables cannot be completely excluded.

An additional limitation is that data on the number of patients at risk at each time point in the survival curves were not available, which may have hindered interpretation of our survival estimates. This is essential for evaluating how attrition and censoring may influence time-to-event outcomes, and without these data, full assessment of the observed survival trends may have been limited.

Although NSBB therapy was accounted for in our propensity matching, the dataset did not distinguish between specific NSBB agents or doses. Due to the absence of these specific data on NSBBs, we were unable to perform a subgroup analysis to investigate carvedilol’s potentially distinct effects relative to other NSBBs.^24^ As a result, the possible influence of specific NSBB therapies on our findings remains uncertain.

Moreover, although our inclusion criteria helped minimize missing data for key baseline variables, we cannot entirely rule out other mechanisms of missingness (eg, not missing at random). Additionally, the federated design of TriNetX is susceptible to electronic health record discontinuity, as patients may receive care outside participating institutions, potentially leading to under-ascertainment of outcomes. These factors could have introduced residual bias and limited the completeness of our data capture. Finally, given that SGLT-2 inhibitors are predominantly indicated for type 2 diabetes and, more recently, heart failure, our findings may not have generalized to patients with cirrhosis who lack these comorbidities.

## Conclusion

In this cohort study of patients with cirrhosis who were receiving furosemide and spironolactone, SGLT-2 inhibitor use was associated with a lower risk of serious liver events, defined as incidence of ascites, variceal development, hyponatremia, or all-cause mortality. These findings suggest that SGLT-2 inhibitors may offer potential liver-related benefits in patients with cirrhosis. Prospective trials are needed to further evaluate their safety and efficacy. Future studies should specifically examine changes in sodium levels following SGLT-2 inhibitor initiation, as well as the incidence of recurrent urinary tract infections and euglycemic diabetic ketoacidosis, given that these are known adverse effects of this drug class. Additionally, research comparing different types and dosing regimens of SGLT-2 inhibitors would provide valuable insights into optimizing treatment for this population.

## Notes

Supplement 1. **eFigure.** Flowchart of Patient Inclusion in the Study Cohort

Supplement 2.
Data Sharing Statement

## References

[1] Nespoux J, Vallon V. Renal effects of SGLT2 inhibitors: an update. Curr Opin Nephrol Hypertens. 2020;29(2):190-198. doi:10.1097/MNH.0000000000000584 31815757 PMC7224333

[2] Fonseca-Correa JI, Correa-Rotter R. Sodium-glucose cotransporter 2 inhibitors mechanisms of action: a review. Front Med (Lausanne). 2021;8:777861. doi:10.3389/fmed.2021.777861 34988095 PMC8720766

[3] Tang J, Ye L, Yan Q, Zhang X, Wang L. Effects of sodium-glucose cotransporter 2 inhibitors on water and sodium metabolism. Front Pharmacol. 2022;13:800490. doi:10.3389/fphar.2022.800490 35281930 PMC8905496

[4] Packer M. Molecular, cellular, and clinical evidence that sodium-glucose cotransporter 2 inhibitors act as neurohormonal antagonists when used for the treatment of chronic heart failure. J Am Heart Assoc. 2020;9(16):e016270. doi:10.1161/JAHA.120.016270 32791029 PMC7660825

[5] Heidenreich PA, Bozkurt B, Aguilar D, . 2022 AHA/ACC/HFSA Guideline for the Management of Heart Failure: Executive Summary: a report of the American College of Cardiology/American Heart Association Joint Committee on Clinical Practice Guidelines. Circulation. 2022;145(18):e876-e894. doi:10.1161/CIR.0000000000001062 35363500

[6] Angeli P, Fasolato S, Mazza E, . Combined versus sequential diuretic treatment of ascites in non-azotaemic patients with cirrhosis: results of an open randomised clinical trial. Gut. 2010;59(1):98-104. doi:10.1136/gut.2008.176495 19570764

[7] Santos J, Planas R, Pardo A, . Spironolactone alone or in combination with furosemide in the treatment of moderate ascites in nonazotemic cirrhosis. a randomized comparative study of efficacy and safety. J Hepatol. 2003;39(2):187-192. doi:10.1016/S0168-8278(03)00188-0 12873814

[8] Biggins SW, Angeli P, Garcia-Tsao G, . Diagnosis, evaluation, and management of ascites, spontaneous bacterial peritonitis and hepatorenal syndrome: 2021 practice guidance by the American Association for the Study of Liver Diseases. Hepatology. 2021;74(2):1014-1048. doi:10.1002/hep.31884 33942342

[9] TriNetX. Accessed August 19, 2024. https://trinetx.com/

[10] Austin PC. An introduction to propensity score methods for reducing the effects of confounding in observational studies. Multivariate Behav Res. 2011;46(3):399-424. doi:10.1080/00273171.2011.568786 21818162 PMC3144483

[11] Normand ST, Landrum MB, Guadagnoli E, . Validating recommendations for coronary angiography following acute myocardial infarction in the elderly: a matched analysis using propensity scores. J Clin Epidemiol. 2001;54(4):387-398. doi:10.1016/S0895-4356(00)00321-8 11297888

[12] VanderWeele TJ, Ding P. Sensitivity analysis in observational research: introducing the E-value. Ann Intern Med. 2017;167(4):268-274. doi:10.7326/M16-2607 28693043

[13] Prasad V, Jena AB. Prespecified falsification end points: can they validate true observational associations? JAMA. 2013;309(3):241-242. doi:10.1001/jama.2012.96867 23321761

[14] Huynh DJ, Renelus BD, Jamorabo DS. Reduced mortality and morbidity associated with metformin and SGLT2 inhibitor therapy in patients with type 2 diabetes mellitus and cirrhosis. BMC Gastroenterol. 2023;23(1):450. doi:10.1186/s12876-023-03085-8 38114915 PMC10731715

[15] Mao X, Zhang X, Kam L, . Synergistic association of sodium-glucose cotransporter-2 inhibitor and metformin on liver and non-liver complications in patients with type 2 diabetes mellitus and metabolic dysfunction-associated steatotic liver disease. Gut. 2024;73(12):2054-2061. doi:10.1136/gutjnl-2024-332481 39122360

[16] Miyamoto Y, Honda A, Yokose S, Nagata M, Miyamoto J. Weaning from concentrated ascites reinfusion therapy for refractory ascites by SGLT2 inhibitor. Clin Kidney J. 2021;15(4):831-833. doi:10.1093/ckj/sfab266 35371450 PMC8967662

[17] Londoño MC, Cárdenas A, Guevara M, . MELD score and serum sodium in the prediction of survival of patients with cirrhosis awaiting liver transplantation. Gut. 2007;56(9):1283-1290. doi:10.1136/gut.2006.102764 17452425 PMC1954951

[18] Guevara M, Baccaro ME, Torre A, . Hyponatremia is a risk factor of hepatic encephalopathy in patients with cirrhosis: a prospective study with time-dependent analysis. Am J Gastroenterol. 2009;104(6):1382-1389. doi:10.1038/ajg.2009.293 19455124

[19] Refardt J, Imber C, Nobbenhuis R, . Treatment effect of the SGLT2 inhibitor empagliflozin on chronic syndrome of inappropriate antidiuresis: results of a randomized, double-blind, placebo-controlled, crossover trial. J Am Soc Nephrol. 2023;34(2):322-332. doi:10.1681/ASN.2022050623 36396331 PMC10103093

[20] Katsuyama H, Hakoshima M, Iijima T, Adachi H, Yanai H. Effects of sodium-glucose cotransporter 2 inhibitors on hepatic fibrosis in patients with type 2 diabetes: a chart-based analysis. J Endocrinol Metab. 2020;10(1):1-7. doi:10.14740/jem632

[21] Tahara A, Kurosaki E, Yokono M, . Effects of SGLT2 selective inhibitor ipragliflozin on hyperglycemia, hyperlipidemia, hepatic steatosis, oxidative stress, inflammation, and obesity in type 2 diabetic mice. Eur J Pharmacol. 2013;715(1-3):246-255. doi:10.1016/j.ejphar.2013.05.014 23707905

[22] Jojima T, Tomotsune T, Iijima T, Akimoto K, Suzuki K, Aso Y. Empagliflozin (an SGLT2 inhibitor), alone or in combination with linagliptin (a DPP-4 inhibitor), prevents steatohepatitis in a novel mouse model of non-alcoholic steatohepatitis and diabetes. Diabetol Metab Syndr. 2016;8(1):45. doi:10.1186/s13098-016-0169-x 27462372 PMC4960737

[23] Hsu SJ, Huang HC, Pun CK, . Sodium-glucose cotransporter-2 inhibition exacerbates hepatic encephalopathy in biliary cirrhotic rats. J Pharmacol Exp Ther. 2022;383(1):25-31. doi:10.1124/jpet.122.001289 35926870

[24] Bañares R, Moitinho E, Piqueras B, . Carvedilol, a new nonselective beta-blocker with intrinsic anti-alpha1-adrenergic activity, has a greater portal hypotensive effect than propranolol in patients with cirrhosis. Hepatology. 1999;30(1):79-83. doi:10.1002/hep.510300124 10385642

