# Supplementary material for: Sodium-Glucose Cotransporter 2 Inhibitors and Serious Liver Events in Patients With Cirrhosis
Source: JAMA Netw Open. 2025 Jun 27;8(6):e2518470. doi: 10.1001/jamanetworkopen.2025.18470 (PMC12205396; doi:10.1001/jamanetworkopen.2025.18470)
Supplement: Supplement 1. — eFigure. Flowchart of Patient Inclusion in the Study Cohort [file jamanetwopen-e2518470-s001.pdf]

Supplemental Online Content

Abu-Hammour MN, Abdel-Razeq R, Vignarajah A, et al. Sodium-glucose cotransporter 2 inhibitors and serious liver events in patients with cirrhosis. *JAMA Netw Open*. 2025;8(7):e2518470. doi:10.1001/jamanetworkopen.2025.18470

eFigure. Flowchart of Patient Inclusion in the Study Cohort

This supplemental material has been provided by the authors to give readers additional information about their work.

© 2025 Abu-Hammour MN et al. *JAMA Network Open*.

eFigure. Flowchart of Patient Inclusion in the Study Cohort

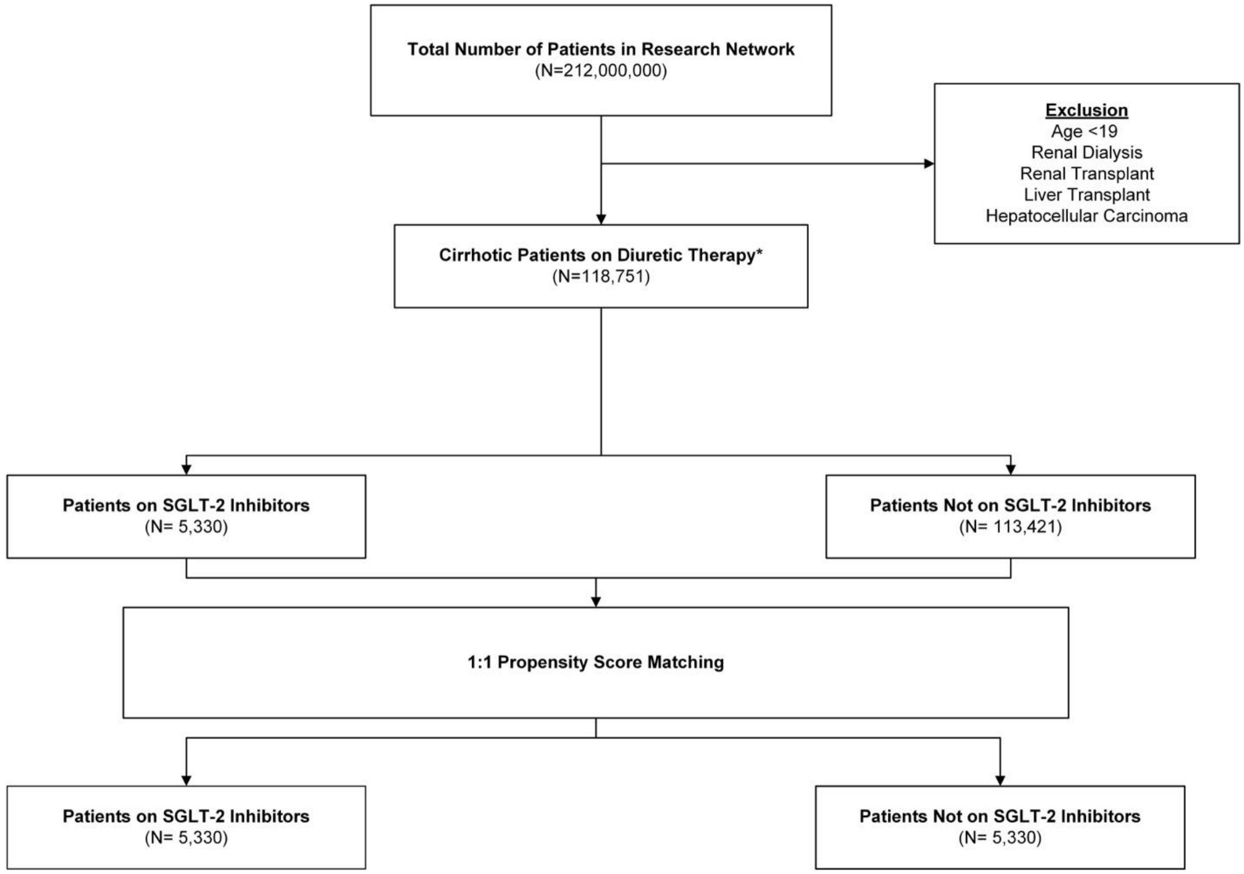

SGLT-2 inhibitors, sodium-glucose cotransporter 2 inhibitors.

*Diuretic Therapy: Furosemide and Spironolactone.

© 2025 Abu-Hammour MN et al. *JAMA Network Open*.
