# Supplementary material for: Sodium-Glucose Cotransporter 2 Inhibitors and Serious Liver Events in Patients With Cirrhosis
Source: JAMA Netw Open. 2025 Jun 27;8(6):e2518470. doi: 10.1001/jamanetworkopen.2025.18470 (PMC12205396; doi:10.1001/jamanetworkopen.2025.18470)
Supplement: Supplement 2. — Data Sharing Statement [file jamanetwopen-e2518470-s002.pdf]

Data Sharing Statement

Abu-Hammour. Sodium-Glucose Cotransporter 2 Inhibitors and Serious Liver Events in Patients With Cirrhosis. *JAMA Netw Open*. Published July 01, 2025. doi:10.1001/jamanetworkopen.2025.18470

Data
Data available: No

Additional Information
Explanation for why data not available: No additional data is available
